# The Poststroke Peripheral Immune Response Is Differentially Regulated by Leukemia Inhibitory Factor in Aged Male and Female Rodents

**DOI:** 10.1155/2020/8880244

**Published:** 2020-12-10

**Authors:** Stephanie M. Davis, Lisa A. Collier, Sarah J. Messmer, Keith R. Pennypacker

**Affiliations:** ^1^Department of Neurology, College of Medicine, University of Kentucky, Lexington, KY 740 S. Limestone, 40536, USA; ^2^Department of Neuroscience, College of Medicine, University of Kentucky, Lexington, KY 800 Rose St., 40536, USA

## Abstract

**Background:**

The goal of this study was to determine whether leukemia inhibitory factor (LIF) promotes anti-inflammatory activity after stroke in a sex-dependent manner.

**Methods:**

Aged (18-month-old) Sprague-Dawley rats of both sexes underwent sham surgery or permanent middle cerebral artery occlusion (MCAO). Animals received three doses of intravenous LIF (125 *μ*g/kg) or PBS at 6, 24, and 48 h before euthanization at 72 h. Spleen weights were measured immediately following euthanization. Western blot was used to measure protein levels of CCL8, CD11b, CXCL9, CXCL10, IL-12 p40, IL-3, and the LIF receptor (LIFR) in spleen tissue. ELISA was used to measure IL-1*β*, IL-6, TNF*α*, and IFN*γ* in spleen tissue. A Griess Assay was used to indirectly quantify NO levels via measurement of nitrite. Levels of cellular markers and inflammatory mediators were normalized to the baseline (sham) group from each sex. Statistical analysis was performed using two-way ANOVA and followed by Fisher's LSD post hoc test.

**Results:**

Aged female rats showed a significantly lower spleen weight after MCAO, but showed a significant increase in spleen size after LIF treatment. This effect was observed in aged male rats, but not to as great of an extent. CD11b levels were significantly higher in the spleens of MCAO+PBS males compared to their female counterparts, but there was no significant difference in CD11b levels between MCAO+LIF males and females. LIF significantly increased CXCL9 after LIF treatment in aged male and female rats. LIFR and IL-3 were upregulated after LIF treatment in aged females. Splenic nitrate increased after MCAO but decreased after LIF treatment in aged females. Splenic nitrate levels did not increase after MCAO but did increase after LIF treatment in aged males. The following cytokines/chemokines were not altered by sex or treatment: TNF*α*, IL-6, IL-12 p40, CCL8, IFN*γ*, and CXCL10.

**Conclusions:**

LIF treatment after permanent MCAO induces sex-dependent effects on the poststroke splenic response and the production of proinflammatory cytokines among aged rats.

## 1. Introduction

Ischemic stroke is currently the fifth leading cause of death in the United States and one of the leading causes of adult disability. The most damaging type of stroke, an emergent large vessel occlusion (ELVO) occurs when a thrombus blocks flow to one of the brain's major arteries [1]. While intravenous tissue plasminogen activator (tPA) is effective at treating small-vessel strokes, it is often ineffective at restoring blood flow during an ELVO [2–4]. ELVO patients may undergo endovascular thrombectomy (EVT), in which a stent retriever is used to retrieve the thrombus from the occluded vessel, up to 24 h after the patient's last known normal [5]. Patients who are ineligible for EVT are extremely limited concerning their treatment options. Furthermore, the prognosis for ELVO without recanalization is extremely dismal; most patients are at risk of death or left with severe deficits in sensorimotor skills and cognition [6].

While acute energy failure is responsible for the initial phase of neuronal death, the majority of tissue damage occurs hours to days after the onset of stroke [7, 8]. Microglia activated by substances released from dying neurons produce  proinflammatory mediators that compromise the blood-brain barrier [9–13]. During this period, increased adrenergic signaling in splenic nerves promotes contraction of the spleen and release of splenocyte populations into the peripheral circulation [14–18]. The breakdown of the blood-brain barrier usually coincides with the production of matrix metalloproteinases by activated microglia [10, 19, 20]. Splenocytes infiltrate the ischemic tissue and further perpetuate the inflammatory cascade through the subsequent release of inflammatory mediators and reactive oxygen species [21]. This poststroke splenic response has been observed in human stroke patients in addition to rodents [22].

Interferon-gamma (IFN*γ*), a proinflammatory cytokine produced by CD4+ T cells, CD8+ T cells, and NK cells, is largely responsible for initiating the peripheral immune response after stroke [23, 24]. Upon stimulation with proinflammatory cytokines such as IL-2 and IL-12, T cells release IFN*γ*, which stimulates the production of CXCL10 in monocytes/macrophages and microglia [25–28]. Offner et al. showed that several proinflammatory chemokines, including CXCL10, are upregulated 22 h after stroke [29]. CXCL10, which increases intracellular calcium through binding to CXCR3, promotes chemotaxis of T cells and other peripheral leukocytes towards the site of injury [30–32]. Administration of antibodies to block the IFN*γ*/CXCL10 signaling yields a significant decrease in the infarct volume and prevents the reduction in spleen size at 72 h after stroke [24]. Furthermore, blocking this signaling pathway significantly reduced the poststroke infiltration of immune response into the brain [23, 24].

Leukemia inhibitory factor (LIF), a cytokine in the IL-6 family, has shown efficacy in promoting cellular survival and countering peripheral inflammation when administered intravenously after permanent stroke [33–37]. LIF activates several signaling pathways, including the PI3K/Akt signaling cascade, which promotes the upregulation of antioxidant enzymes including metallothionein III and peroxiredoxin IV in oligodendrocytes and superoxide dismutase 3 in neurons [34, 35]. Also, LIF treatment promotes anti-inflammatory signaling by decreasing splenic levels of IFN*γ* and preventing the upregulation of CXCL10 that occurs after stroke [36]. However, these studies were performed in 3-month-old male rats, which are equivalent age-wise to adolescent male humans. Considering that the risk of stroke doubles with every decade after the age of 55, the use of young animals of one sex to mimic human stroke patients is not an optimal model.

Recently, this group demonstrated that administration of LIF at 6, 24, and 48 h after MCAO promotes partial motor skill recovery in aged female rats, but does not significantly decrease tissue damage [38]. This phenomenon is attributed to the significantly lower levels of LIFR in the brain tissue of aged males and females compared to young male rats. However, very little is known about the role of the aged immune system in poststroke pathophysiology. This manuscript explores how LIF treatment modulates the splenic inflammatory response after stroke in an age-dependent and sex-dependent manner.

## 2. Materials and Methods

Retired breeding pairs of Sprague-Dawley rats (9-10 mo.) were purchased from Envigo (Indianapolis, IN) and housed until they reached the age of 18-19 mo. All animals were kept in a climate-controlled room and allowed access to food and water *ad libitum*. Animals were kept on a 12 h light/dark cycle (07:00–19:00 h). All animals were randomly assigned to treatment groups before surgical procedures, and sample sizes were calculated using StatMate software (GraphPad, San Diego, CA). All procedures were approved according to the University of Kentucky Institutional Animal Care and Use Committee.

### 2.1. Middle Cerebral Artery Occlusion

Permanent focal cerebral ischemia was induced using the intraluminal middle cerebral artery occlusion (MCAO) model as previously described [39]. A 40 mm monofilament was introduced through the ligated external carotid artery and advanced through the internal carotid artery. The filament was advanced until it reached the origin of the middle cerebral artery. Animals subjected to the sham MCAO procedure were anesthetized and underwent exposure of the common carotid artery without subsequent occlusion of the middle cerebral artery.

### 2.2. Drug Treatment

All animals were treated prophylactically with ketoprofen (10 mg/kg s.c.) and atropine (0.25 mg/kg s.c.) with two additional doses of ketoprofen at 24 and 48 h post-MCAO. Recombinant human LIF (ProSpec, Ness Ziona, Israel) (125 *μ*g/kg) or PBS (pH 7.4) was administered intravenously at 6, 24, and 48 h post-MCAO as previously described [34, 35]. Animals were randomly assigned to treatment groups, and all lab personnel administering drugs were blinded to treatments.

### 2.3. Tissue Collection

Rats were euthanized at 72 h post-MCAO via intraperitoneal injection of ketamine/xylazine solution (75 mg/kg and 7.5 mg/kg) [23]. Spleens were collected immediately before perfusion. Animals used for biochemical analysis were perfused with saline before obtaining tissue.

### 2.4. Tissue Homogenization

To obtain whole-cell extracts, frozen tissue was homogenized in a whole-cell lysis buffer containing the following: 50 mM Tris pH 8, 150 mM NaCl, 0.1% SDS, 1% IGEPAL, 1 mM PMSF, and a complete Mini protease inhibitor cocktail (Roche Diagnostics, Indianapolis, IN). An electric homogenizer was used to disrupt tissue, and lysates were incubated on ice for 20 min. Tissue lysates were vortexed and pipetted to break up nuclei. Protein concentrations were determined by performing a Bradford Assay according to the manufacturer's protocol (Bio-Rad, Hercules, CA). Briefly, a Bradford reagent containing Coomassie blue was added to the diluted protein samples, and absorbance was read at 595 nm using a SmartSpec 3000 spectrophotometer (Bio-Rad). Concentrations were determined by comparing the absorbance readings against a standard curve.

### 2.5. Western Blot

Western blot analysis was used for semiquantitative measurement of protein expression using a previously described procedure [35]. Briefly, whole-cell lysates from spleen tissue were run on SDS-PAGE gels and transferred to nitrocellulose membranes. Membranes were blocked with 5% nonfat milk in TBS and probed with the following antibodies: rabbit anti-LIFR (C-19) (1 : 100; Santa Cruz Biotechnology, Dallas, TX, US), rabbit anti-IL-12 p40 (1 : 100; ABBiotech, San Diego, CA), rabbit anti-CXCL10 (1 : 5000; Abcam, San Francisco, US), rabbit anti-IL-3 (R&D Biosystems; Minneapolis, MN), rabbit anti-CXCL9 (1 : 1000; Fitzgerald Industries Int., North Action, MA), rabbit-anti-CCL8 (1 : 500; Biorbyt, Cambridge, UK), and mouse anti-CD11b (1 : 1000; Abcam). Membranes were incubated in IRDye 800CW goat anti-rabbit antibodies (1 : 20,000; Li-Cor) for the detection of protein bands. Membranes were visualized using the Odyssey CLx Imaging System (Li-Cor). To normalize for loading, membranes containing whole-cell extracts were reprobed with mouse anti-*β*-actin (1 : 5000; Novus Biologicals) and IRDye 680RD goat anti-mouse antibodies (1 : 20,000; Li-Cor). Western blot bands were quantified using ImageJ software (NIH, Bethesda, MD).

### 2.6. Griess Assay

For indirect measurement of NO production, a Griess Assay Kit (Molecular Probes, Eugene, OR), was used to measure nitrite (NO_2_^−^) levels in the spleen tissue from aged rats. The assay was performed according to the manufacturer's protocol. The concentration of NO_2_^−^ in each spleen sample was normalized to the protein concentration and expressed in terms of % baseline (sham) levels.

### 2.7. ELISA

To measure the release of IFN*γ*, TNF*α*, IL-1*β*, IL-6, and IL-10 in spleen tissue, ELISA was performed as previously described according to the manufacturer's protocol using the appropriate DuoSet ELISA kits (R&D Systems, Inc., Minneapolis, MN). The concentration of the cytokine in each spleen sample was normalized to the protein concentration and expressed in terms of % baseline (sham) levels.

### 2.8. Data Analysis

All data are expressed as the mean ± the standard error of the mean. All data from treatment groups were normalized to the mean of the respective sham group from each sex. Statistical analysis was performed using GraphPad Prism 8.4.3. Software (La Jolla, CA). Data were analyzed using a two-way ANOVA followed by Fisher's protected LSD post hoc test to determine statistical significance. If a significant interaction was found within the two-way ANOVA, individual differences were determined using Fisher's protected LSD post hoc test. A *p*-value of less than 0.05 was considered statistically significant. All reported *p*-values are two-tailed.

## 3. Results

### 3.1. LIF Treatment Increases Spleen Weight after MCAO in Aged Animals

**Figure 1:**
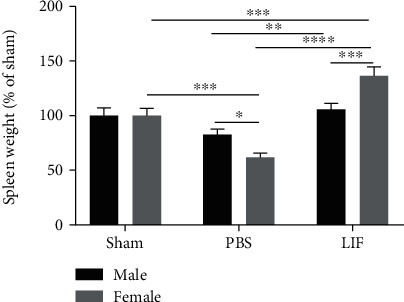
LIF treatment increases spleen weight after MCAO in aged animals. Spleen weights were measured after euthanization and normalized to the average spleen weight for sham-operated male (*n* = 9) and female animals (*n* = 8). The average spleen size among LIF-treated males (*n* = 23) was significantly higher than that among PBS-treated males (*n* = 19; ^∗∗^*p* < 0.01), but not sham males. Although there was a significant decrease in spleen size after MCAO among female rats (^∗∗∗^*p* < 0.001), spleens from LIF-treated female rats were significantly larger than spleens from sham (^∗∗∗^*p* < 0.001) and MCAO+PBS females (^∗∗∗∗^*p* < 0.0001). Furthermore, female MCAO+PBS rats (*n* = 14) showed a significantly larger decrease in spleen weight compared to PBS-treated males (^∗^*p* < 0.05). However, LIF-treated females (*n* = 16) had significantly larger spleens compared to their male counterparts (^∗∗∗^*p* < 0.001). Male sham *n* = 8‐24 per treatment group.

Spleen weights were measured immediately following euthanization at 72 h after MCAO and normalized to the mean spleen weight from aged male and aged female rats. There was a significant effect of drug treatment on spleen weight (*F*_2,83_ = 38.07; *p* < 0.0001) as well as a significant interaction between sex and treatment (*F*_2,83_ = 10.71; *p* < 0.0001). While there was a significant decrease in spleen size among female MCAO+PBS rats compared to sham females (*p* < 0.001), the MCAO+LIF females had significantly larger spleens than the MCAO+PBS females (*p* < 0.0001) and sham females (*p* < 0.001). MCAO+PBS males did not show significant changes in spleen size compared to their sham counterparts, but MCAO+LIF males had significantly larger spleens compared to their MCAO+PBS counterparts (*p* < 0.01). Furthermore, while MCAO+PBS females showed a significantly larger decrease in spleen weight compared to their male counterparts (*p* < 0.05), the MCAO+LIF females showed a significantly larger increase in spleen size after treatment compared to their male counterparts (*p* < 0.001; Figure 1).

### 3.2. Sex and Treatment Affect Splenic LIFR and CD11b Expression

**Figure 2:**
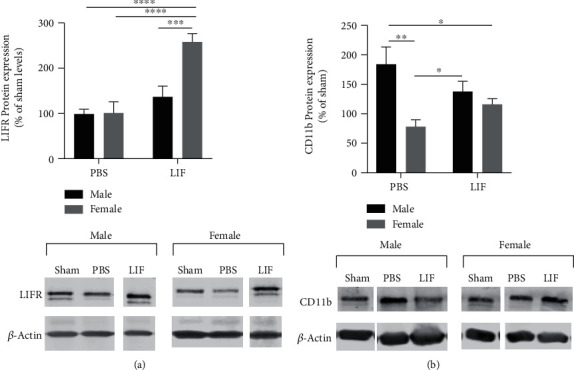
Sex and treatment affect splenic LIFR and CD11b expression. (a) LIFR levels in the spleens from LIF-treated females (*n* = 5) were significantly higher than those in the spleens from PBS-treated females (*n* = 8; ^∗∗^*p* < 0.01). However, there was no significant difference between the spleen weights from LIF-treated male rats (*n* = 8) and the spleen weights from PBS-treated males (*n* = 7). *n* = 3 for both sham groups. (b) CD11b levels were significantly higher in the spleens of PBS-treated male rats (*n* = 4) compared to PBS-treated females (*n* = 4; ^∗∗^*p* < 0.01) and LIF-treated females (*n* = 4). However, there was was no significant difference between LIF-treated males (*n* = 4) and LIF-treated females. *n* = 2 for both sham groups.

Western blot was used to measure levels of LIFR in the spleens of aged rats at 72 h after MCAO. Levels of LIFR protein expression were normalized to respective sham levels from each sex. There were significant effects of sex (*F*_1,24_ = 7.931; *p* = 0.0096) and treatment (*F*_1,24_ = 19.79; *p* = 0.0002) on LIFR protein expression, as well as a significant interaction between the two factors (*F*_1,24_ = 7.455; *p* = 0.0117). Spleens from LIF-treated females showed significantly higher levels of relative LIFR expression compared to PBS-treated animals of both sexes (*p* < 0.0001) and MCAO+LIF males (*p* < 0.001). However, there was no significant difference between the spleen weights from LIF-treated males and PBS-treated males (Figure 2(a)). To determine levels of CD11b in the spleen after stroke, spleen samples from animals euthanized 72 h after MCAO were analyzed via western blot. Levels of relative CD11b expression in treatment groups were normalized to those in sham groups of the same sex. There was a significant effect of sex on CD11b expression (*F*_1,12_ = 11.73; *p* = 0.0050) and a significant interaction between sex and treatment (*F*_1,12_ = 5.055; *p* = 0.0441). Relative CD11b expression was significantly lower in the MCAO+PBS female rats compared to MCAO+PBS males (*p* < 0.01) and MCAO+LIF males (*p* < 0.05). Furthermore, CD11b levels were significantly higher in MCAO+PBS males compared to MCAO+LIF females (*p* < 0.05). However, there was no significant difference in CD11b levels between LIF-treated males and females (Figure 2(b)).

### 3.3. Sex Affects Splenic IL-1*β*, but Not TNF*α* or IL-6

**Figure 3:**
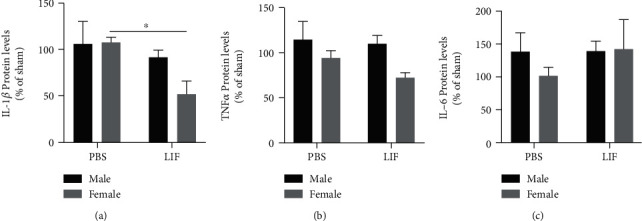
IL-1*β*, but not TNF*α* or IL-6, is affected by sex and treatment. (a) Among LIF-treated rats, aged females (*n* = 5) had significantly lower levels of IL-1*β* compared to their PBS-treated counterparts (*n* = 5; ^∗^*p* < 0.05). However, there was no difference between the relative IL-1*β* expression between PBS-treated males (*n* = 7) and LIF-treated males (*n* = 11). *n* = 4 for both sham groups. However, (b) TNF*α* levels were not significantly altered among PBS-treated males (*n* = 7), LIF-treated males (*n* = 10), PBS-treated females (*n* = 5), and LIF-treated females (*n* = 3). *n* = 3 for male sham and *n* = 4 for female sham groups. (c) Furthermore, IL-6 levels were not significantly altered among PBS-treated males (*n* = 8), LIF-treated males (*n* = 11), PBS-treated females (*n* = 6), and LIF-treated females (*n* = 3). *n* = 3 for both sham groups.

ELISA was used to measure levels of IL-1*β* in spleen tissue from aged males and females at 72 h after stroke. There was a significant effect of treatment on IL-1*β* (*F*_1,24_ = 5.251; *p* = 0.0310), but not due to sex. IL-1*β* levels were significantly higher in the spleens of MCAO+PBS females compared to MCAO+LIF female rats (*p* < 0.05; Figure 3(a)). TNF*α* levels in spleen tissue were also measured in the spleens of aged male and female rats using ELISA. However, there were no significant effects due to sex or drug treatment on splenic TNF*α* levels (Figure 3(b)). IL-6 levels in splenic tissue were also measured using ELISA; however, there were no significant sex- or treatment-dependent alterations in IL-6 levels (Figure 3(c)).

### 3.4. IL-12 p40 and CCL8 Levels Are Unchanged after MCAO+LIF Treatment

**Figure 4:**
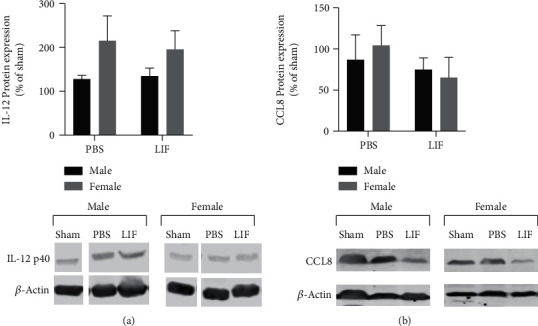
Sex and treatment do not alter IL-12 p40 or CCL8. (a) There were no significant effects of sex or drug treatment on IL-12 p40 levels in the spleens of MCAO+PBS male (*n* = 4) and female rats (*n* = 4). Furthermore, there were no significant changes in relative p40 levels after MCAO+LIF treatment in male (*n* = 4) and female (*n* = 4) rats. *n* = 2 for both sham groups. (b) There were no significant effects of sex or drug treatment on CCL8 levels in the spleens of MCAO+PBS male (*n* = 5) and female rats (*n* = 5). Furthermore, there were no significant changes in relative p40 levels after MCAO+LIF treatment in male (*n* = 5) and female (*n* = 5) rats per treatment group. *n* = 4 for both sham groups.

Western blot was used to measure levels of IL-12 p40 and CCL8 in the spleens of aged male and female rats after MCAO and LIF treatment. There were no significant changes in IL-12 p40 levels due to sex or drug treatment (Figure 4(a)). Furthermore, there were no significant effects of sex or treatment on relative CCL8 levels in spleen tissue (Figure 4(b)).

### 3.5. MCAO+LIF Changes Levels of IFN*γ*-Inducible Inflammatory Mediators but Not IFN*γ*

**Figure 5:**
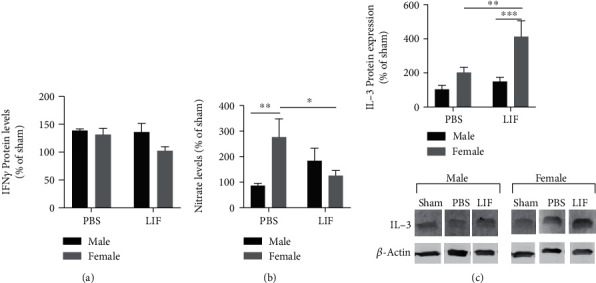
Sex and treatment change levels of IFN*γ* and IFN*γ*-inducible inflammatory mediators. (a) There was no significant difference in IFN*γ* levels in the spleens of male or female rats after MCAO. *n* = 4 per treatment group for all groups except sham groups (*n* = 2). (b) Relative levels of NO_2_^−^ were significantly higher among MCAO+PBS females (*n* = 7) compared to MCAO+PBS males (*n* = 10). LIF significantly decreased NO_2_^−^ in the spleens of female rats (*n* = 8) compared to the MCAO+PBS female rats (*n* = 7; ^∗^*p* < 0.05). There was no significant difference in relative NO_2_^−^ levels between MCAO+LIF male rats (*n* = 6) and MCAO+PBS male rats. *n* = 5 for male sham and *n* = 4 for female sham groups. (c) MCAO+LIF-treated female rats (*n* = 5) had significantly higher levels of IL-3 in the spleen compared to MCAO+PBS females (*n* = 6; ^∗∗^*p* < 0.01) and MCAO+LIF males (*n* = 8; ^∗∗∗^*p* < 0.001). However, there were no differences between MCAO+PBS males (*n* = 7) and MCAO+LIF males. *n* = 2 for male sham group and *n* = 5 for female sham group.

IFN*γ* levels were measured in the spleens of aged rats using an ELISA kit. There were no significant changes in normalized IFN*γ* levels due to sex or treatment (Figure 5(a)). Nitric oxide production was indirectly measured by detecting levels of NO_2_^−^ in spleen tissue using a Griess Assay. The concentration of nitrite was normalized to mM NO_2_^−^ per mg of protein, and all treatment groups were further normalized to those of their respective sham groups. Although the main effects of sex and treatment on NO_2_^−^ were not significant, there was a significant interaction between these two factors (*F*_1,27_ = 10.08; *p* = 0.0037). The MCAO+PBS female had significantly higher levels of NO_2_^−^ compared to male MCAO+PBS rats (*p* < 0.01). However, LIF treatment significantly reduced NO_2_^−^ in female rats compared to their MCAO+PBS counterparts (*p* < 0.05; Figure 5(b)). IL-3 was measured in spleen tissue using western blot. There were significant effects due to sex (*F*_1,22_ = 17.87; *p* = 0.0003) and treatment (*F*_1,22_ = 8.946; *p* = 0.0067). There was no difference between male and female MCAO+PBS rats in terms of splenic IL-3. However, levels of IL-3 in the spleens of MCAO+LIF females were significantly higher than those of MCAO+PBS females (*p* < 0.01) and MCAO+LIF male rats (*p* < 0.001; Figure 5(c)).

### 3.6. LIF Affects Splenic CXCL9, but Not CXCL10 Levels

**Figure 6:**
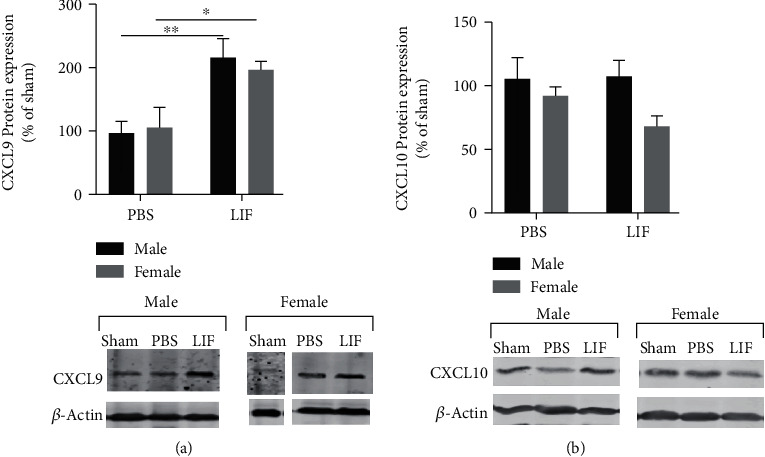
LIF affects splenic CXCL9, but not CXCL10, levels. (a) There was a significant increase in splenic CXCL9 among MCAO+LIF rats (*n* = 4) compared to MCAO+PBS rats (*n* = 4; ^∗∗^*p* < 0.01). Furthermore, LIF treatment increased CXCL9 expression in female rat (*n* = 3) compared to MCAO+PBS female (*n* = 4; ^∗^*p* < 0.05) levels. *n* = 2 for both sham groups. (b) There was no significant change in splenic CXCL10 levels due to treatment or sex after MCAO. *n* = 4 for all treatment groups except both sham groups (*n* = 2).

The IFN*γ*-inducible chemokines CXCL9 and CXCL10 were measured in spleen tissue using western blot. The levels of CXCL9 in the spleen were significantly affected by treatment (*F*_1,11_ = 19.17; *p* = 0.0011) but not by sex. Specifically, there was a significant increase in CXCL9 in the spleens of MCAO+LIF male rats compared to MCAO+PBS male rats (*p* < 0.01), and the MCAO+LIF female rats had significantly higher levels of CXCL9 compared to MCAO+PBS female rats (*p* < 0.05; Figure 6(a)).

Previously, this lab demonstrated that LIF prevents the stroke-mediated increase in splenic CXCL10 at 72 h after stroke. However, among aged males and female rats, there were no sex- or treatment-dependent changes in CXCL10 after MCAO and LIF treatment (Figure 6(b)).

## 4. Discussion

Several published studies have demonstrated that blocking the spleen-mediated immune response can significantly reduce the infiltration of immune cells and subsequent brain damage after stroke [16, 17, 40]. For example, Seifert et al. showed that administration of antibodies against IFN*γ* after stroke significantly reduces tissue damage at 72 h after stroke [23, 24]. Other studies involving the administration of therapies such as human umbilical cord blood cells [21, 41] and the sigma receptor agonist 1,3-Di-o-tolylguanidine show that blocking the splenic response is associated with positive outcomes and reduced brain damage after MCAO.

Although the spleen weights are significantly increased after LIF treatment in both male and female aged rats, the results of this study indicate that female rats show a more robust increase in spleen weight compared to their male counterparts. A previous study by this laboratory showed that administration of LIF at 24, 48, and 72 h after stroke did not significantly decrease edema or cytotoxicity, although there was a trend towards decreased brain damage among female LIF-treated rats. However, aged female rats demonstrated partial motor skill recovery according to two out of the four assessments, while their male counterparts did not show recovery according to any of the four tests. Since levels of LIFR in the brains of aged female rats were lower than those of aged male rats after stroke, the partial recovery observed in aged female rats after LIF treatment may be attributed to stronger anti-inflammatory activity [38].

Among young male rats, CD11b expression significantly decreased after MCAO and was not affected by LIF treatment [36]. Most likely, this pattern of expression is due to the migration of CD11b+ cells from the spleen to the peripheral circulation after stroke. This hypothesis is consistent with what we observed between male and female MCAO+PBS animals; the female animals had significantly lower levels of relative splenic CD11b expression as well as significantly smaller spleens compared to their male counterparts. CD11b levels were significantly higher in the spleens of MCAO+LIF females compared to their PBS-treated counterparts, which reflects the increased spleen weight observed after LIF treatment. However, the increase in spleen size after LIF treatment is much more pronounced than the increase in CD11b levels, which suggests that cell surface expression of CD11b may decrease after LIF treatment. Since CD11b surface expression is an indicator of an inflammatory phenotype [42], its downregulation would suggest anti-inflammatory activity. Further studies using flow cytometry will be necessary to confirm the specific effects of LIF on the cell surface expression of CD11b in immune cells.

The larger increase in spleen size observed after LIF treatment in female rats may also be explained by greater expression of LIFR on splenocytes seen in female rats after LIF treatment. The upregulation of splenic LIFR could be due to a higher number of LIFR+ cells returning to the spleen after LIF treatment. While LIF causes an increase in spleen size in both male and female rats, only females show an upregulation of splenic LIFR after MCAO+LIF treatment. These results also demonstrate that male rats show a differential response in LIFR expression between young males and aged males; according to a previous study from our lab, young males show decreased LIFR after LIF treatment while aged males show no significant change in splenic LIFR levels. However, the increase in spleen size after LIF treatment in aged male rats might obscure any changes in the number of LIFR+ splenocytes, since young male rats do not show a significant change in spleen size after LIF treatment [36].

Previously, this laboratory demonstrated that LIF treatment significantly reduced splenic IFN-gamma and prevented the upregulation of CXCL10 after stroke [36]. Seifert et al. demonstrated that the IFN*γ*/CXCL10 axis is crucial for promoting the chemotaxis of immune cells, specifically CD3+CD4+ Th1/Th17 and CD3+CD8+ cytotoxic T cells, to the infarct [18, 23, 24]. Although IFN*γ* levels were not changed after MCAO or LIF treatment, LIF significantly decreased the concentration of NO_2_^−^, which is a byproduct of NO breakdown, in the spleens of aged female rats but not aged male rats. Decreased NO_2_^−^ levels are likely due to the changes in inducible nitric oxide synthase (iNOS) activity and subsequent NO production [43, 44]. IFN*γ* released from T cells [25] and NK cells [45] increases iNOS expression in monocytes and neutrophils [46], which promotes NO production and the accumulation of NO_2_^−^. Although relative IFN*γ* levels were not significantly decreased after LIF treatment in female rats, iNOS expression may be mediated together by IL-1*β* and IFN*γ* [47]. Therefore, the lower NO_2_^−^ levels observed in spleens of female rats are attributed to the sex-dependent effect of LIF on IL-1*β* production. Sex-specific differences in the production of other proinflammatory cytokines such as IL-1*β* might be responsible for the differential effect of LIF on IFN*γ* levels in the spleen. Posma et al. previously showed that males have higher numbers of IL-1*β*-producing monocytes in the blood compared to females due to higher blood levels of testosterone [48].

Although LIF treatment prevented the increase in splenic CXCL10 in young male rats, we did not observe a significant change in splenic CXCL10 levels after a stroke or LIF treatment among aged rats of either sex [36]. The return of CXCL10+ splenocytes to the spleen after LIF treatment may have obscured any changes in its intracellular expression. However, we did observe a significant upregulation in the cytokine IL-3 and the IFN*γ*-inducible chemokine CXCL9 after MCAO+LIF treatment in aged animals of both sexes. Although the higher levels of these cytokines might be explained by a higher number of CXCL9-producing monocytes and IL-3-producing T cells in the spleen, upregulation of chemotactic cytokines may provide a beneficial role after stroke by drawing inflammatory cells away from the brain [49]. For instance, HUCB cells cause splenocytes to return to the spleen after stroke via the secretion of potent chemotactic cytokines [49, 50].

CXCL9, alternatively known as monokine induced by IFN*γ*, is a potent inducer of T cell chemotaxis that signals through CXCR3 [51]. Therefore, the increase in spleen size observed after LIF treatment in both sexes might be the result of CXCL9-mediated chemotaxis of T cells from the peripheral circulation back to the spleen. Little is known about the role of IL-3 in ischemic stroke; however, it is produced by activated CD4+/CD8+ T cells and has been implicated in the development of neuroinflammatory conditions such as multiple sclerosis [52, 53]. Although IL-3 is mainly a chemotactic factor for eosinophils [54], it has also been shown to regulate the expression of CXCL9 [55] and act synergistically with the chemokine CXCL12 to promote T cell chemotaxis. Notably, Renner et al. demonstrated that treatment with anti-IL-3 antibodies decreased leukocytic infiltration among patients with relapsing-remitting multiple sclerosis. Since IL-3 is secreted by T cells [56], the higher level of IL-3 and CXCL9 observed after LIF treatment could also be explained by a higher number of T cells in the spleen.

The results of this study demonstrate the necessity of using age-appropriate animal models that take sex as a biological variable into account. Preclinical and clinical studies testing the efficacy of potential anti-inflammatory and neuroprotective treatments for stroke demonstrate that certain drugs are efficacious in male, but not female, patients and vice versa. For instance, minocycline, an antibiotic and poly-ADP ribose polymerase inhibitor, only demonstrated neuroprotective efficacy in male mice after ischemic stroke, but not ovariectomized female mice [57]. A subsequent clinical study mirrored the sex-dependent effects of the initial preclinical study; while male patients showed recovery after minocycline administration, there was no observable effect in female patients [58]. A clinical study using uric acid as a neuroprotective agent against ischemic stroke is further evidence of the need to take sex as a biological variable into account when examining poststroke outcomes. While initial results suggested that uric acid did not improve outcomes, further analysis of the data showed that female patients experienced positive outcomes after treatment [59]. Our previous study, which showed that aged female rats, but not aged male rats, showed motor skill recovery after LIF treatment, further demonstrates the need to examine neuroprotective therapeutics in age-appropriate models of both sexes [38].

Although this study provided insight regarding the effect of LIF on the poststroke immune response in aged male and female animals, some limitations need to be discussed. While western blot analysis and ELISA demonstrate relative changes in levels of inflammatory mediators compared to baseline (sham) and PBS treatment, it does not provide inflammation on cell-specific level markers such as CD11b/LIFR. Furthermore, western blots do not provide information about which cell types are responsible for expressing these mediators. Therefore, future studies using flow cytometry and immunohistochemistry will be necessary to confirm the anti-inflammatory mechanisms of LIF in aged animals and determine which splenocyte populations are the most responsive to LIF treatment.

## 5. Conclusion

Through these experiments, we have shown that LIF exerts stronger anti-inflammatory effects on the poststroke splenic response in aged female rats, but not male rats. We believe these effects were due to the upregulation of chemotactic cytokines as well as the downregulation of inflammatory mediators such as NO and IL-1*β* in the splenocytes of aged female rats. Future studies using flow cytometry will be used to determine whether higher levels of cytokines such as CXCL9 and IL-3 are due to higher numbers of T cells and monocytes/macrophages with the spleen or higher production by each cell.

## Data Availability

All data generated and analyzed during this study are not publicly available but can be made available from the corresponding author upon request.
